# Caught between fear and tradition: parental knowledge, beliefs and emergency responses to paediatric snakebites in rural Sri Lanka

**DOI:** 10.1136/bmjpo-2025-003658

**Published:** 2025-06-13

**Authors:** Kavinda Dayasiri, Tharuka Perera, Indika Gawarammana, Shaluka Jayamanne

**Affiliations:** 1 University of Kelaniya, Kelaniya, Sri Lanka; 2 University of Peradeniya, Peradeniya, Sri Lanka

**Keywords:** Child Health, Developing Countries, Epidemiology, Health services research, Toxicology

## Abstract

**Background:**

Paediatric snakebite remains a critical yet underexplored public health issue in rural Sri Lanka, where children are particularly vulnerable due to ecological exposure, limited access to timely care and entrenched traditional beliefs. While biomedical advancements exist, parental knowledge, cultural practices and systemic barriers significantly shape prehospital responses and outcomes.

**Methods:**

This qualitative exploratory study employed 10 focus group discussions with 70 parents (45 mothers, 25 fathers) from snakebite-endemic rural communities in the Ampara and Polonnaruwa Districts. Participants were purposively selected, including those with direct or indirect exposure to snakebite incidents. Data were collected using a semistructured guide and analysed thematically, following Braun and Clarke’s six-phase framework. Themes were validated through member checking and intercoder agreement.

**Results:**

Five interrelated themes emerged: (1) pervasive fear and psychological burden associated with snakebite risk, (2) fragmented and inconsistent knowledge of envenomation symptoms and first-aid, (3) environmental and structural vulnerabilities such as unsafe housing and proximity to snake habitats, (4) strong adherence to traditional beliefs and ritual practices and (5) a high demand for culturally tailored education and systemic support. Many parents employed harmful first-aid methods due to inherited practices and a lack of formal training. Despite challenges, participants expressed strong willingness to learn and adopt evidence-based strategies.

**Conclusions:**

Effective paediatric snakebite prevention and management require context-specific, culturally sensitive interventions that address both knowledge gaps and structural barriers. Engaging with local belief systems and improving healthcare accessibility are essential for reducing snakebite-related morbidity and mortality among children in rural Sri Lanka.

**WHAT IS ALREADY KNOWN ON THIS TOPIC**

- Snakebites are a significant public health concern in rural Sri Lanka, with children being particularly vulnerable.
- Parental attitudes, knowledge and preventive practices play a crucial role in mitigating snakebite risks.
- Limited awareness and reliance on traditional beliefs can influence healthcare-seeking behaviour and first-aid responses.

**WHAT THIS STUDY ADDS**

- Identifies key determinants of parental attitudes towards snakes and snakebites, including fear, first-aid training and socioeconomic factors.
- Highlights gaps in parental knowledge and preventive practices.
- Demonstrates that social media is the primary source of snakebite-related information for parents.

**HOW THIS STUDY MIGHT AFFECT RESEARCH, PRACTICE OR POLICY**

- Emphasises the need for targeted educational programmes to improve snakebite-related attitudes among parents.
- Supports the development of community-based interventions to address misconceptions and enhance preventive measures.
- Provides evidence for policymakers to integrate structured snakebite education into maternal and child healthcare initiatives.

## Introduction

 Snakebite envenomation remains a neglected tropical disease with substantial public health consequences in rural and resource-limited regions, particularly across South Asia.^1^ Sri Lanka has one of the highest incidence rates of snakebite globally, with an estimated 30 000–40 000 cases annually.^2^ While adults, especially those engaged in agriculture, constitute a significant proportion of victims, paediatric snakebites are increasingly recognised as a critical yet underaddressed issue.^3^ Children are more vulnerable to severe envenomation due to their smaller body size, and delays in first aid or hospital transfer can prove fatal.^4^ Despite growing biomedical advances in snakebite treatment, prehospital care and community-level responses remain poorly understood, especially from the perspective of caregivers in endemic areas.^5^

In rural Sri Lanka, the burden of paediatric snakebite is compounded by structural, educational and cultural factors.^6^ Households often lack protective infrastructure, access to emergency transport and timely medical care. Moreover, traditional beliefs, inherited practices and cultural narratives about snakes influence how caregivers perceive, interpret and respond to snakebite incidents. Parents play a central role in the prehospital response, including recognition of symptoms, administration of first aid and decisions around whether and when to seek biomedical treatment. Yet, their experiences, anxieties and decision-making processes remain insufficiently documented in the academic literature. This study aimed to explore the knowledge, beliefs and emergency responses of parents living in snakebite-prone rural communities in Sri Lanka, with a specific focus on the psychosocial and systemic challenges they face in preventing and managing paediatric snakebites.

## Methods

### Study design

This study adopted a qualitative exploratory design, employing focus group discussions (FGDs) to elicit in-depth insights into the experiences and challenges faced by parents of children living in regions with a high burden of snakebite in Sri Lanka. The inquiry specifically aimed to explore persistent parental anxiety, psychosocial impacts, awareness of clinical symptoms and first-aid practices, environmental and structural risk factors, reliance on traditional treatment modalities, and openness to health education and systemic interventions.

### Study setting and participants

**Figure 1:**
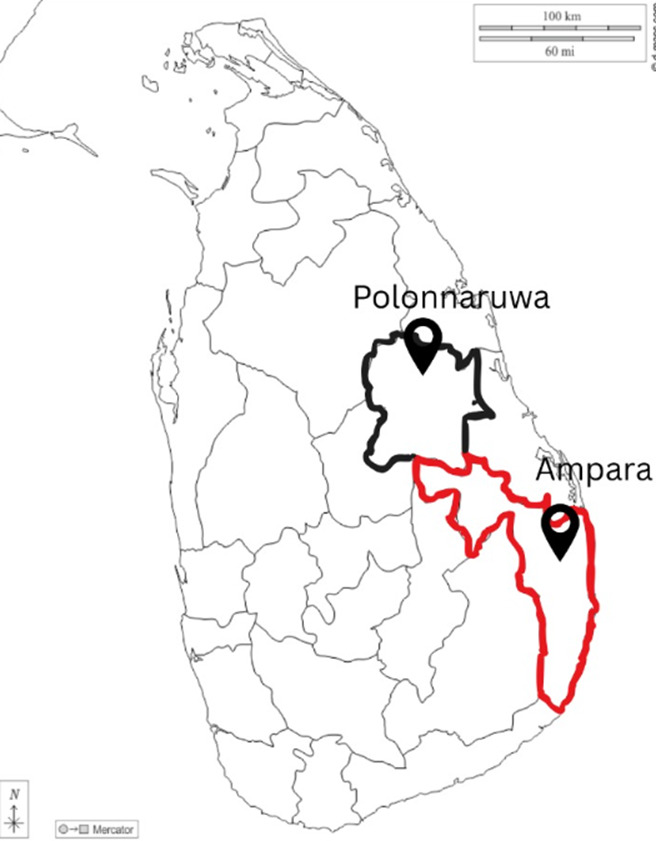
Geographical distribution of study settings.

The study was conducted in the rural settings of the Ampara and Polonnaruwa Districts, which were purposively selected based on national epidemiological data identifying them as high-incidence zones for medically significant snakebite envenomation.^7^ These regions consistently report a disproportionately high number of hospital admissions due to snakebites, with a significant proportion of cases involving children.^8^ Both districts are predominantly agrarian, and frequent human-snake encounters occur due to occupational activities and residential proximity to natural snake habitats. A total of 70 parents (45 mothers and 25 fathers), all permanent residents of these districts with at least one child under the age of 5 years, participated in the study (Figure 1).

### Sampling strategy

A purposive sampling strategy was employed to ensure the relevance and depth of the data collected. Recruitment was coordinated through Public Health Midwives working in local health divisions, who identified and approached potential participants based on predefined inclusion criteria. Participants were selected to reflect a range of experiences related to paediatric and community-level snakebite exposure. Of the 70 participants, 20 parents were specifically selected due to direct or indirect exposure to snakebite incidents—either involving their own child, a family member, or another individual within their village. All eligible individuals invited to participate consented to the study and attended their scheduled focus group sessions; there were no refusals or participant dropouts.

### Facilitator characteristics and reflexivity

All FGDs were moderated by the principal investigator, a male physician with over 15 years of clinical experience and a specialised background in paediatric toxicology and qualitative health research. He was supported by a trained male assistant moderator with expertise in public health and qualitative methodologies. Both facilitators had undergone formal instruction in qualitative interviewing techniques. The principal investigator had no prior professional or personal relationships with any participants, minimising potential bias and ensuring neutrality in the discussion process.

### Data collection procedures

Ten FGDs were conducted, each comprising six to eight participants to maintain group manageability and facilitate inclusive discussion. Sessions lasted approximately 30–45 min and were conducted in community settings conducive to privacy and open dialogue. A semistructured interview guide, developed around five core thematic areas, was pilot-tested with a small group of parents (n=5) from a neighbouring locality to refine clarity and cultural sensitivity.

The facilitation strategy emphasised participant-led narratives, with open-ended prompts designed to elicit unfiltered lived experiences. The core domains included: (1) parental fear and psychological burden, (2) recognition of snakebite symptoms and knowledge of first-aid, (3) environmental and housing-related risk factors, (4) cultural and traditional treatment practices and perceived utility of community education and systemic support. All discussions were conducted in participants’ native language, audio-recorded with prior informed consent and supplemented by field notes documenting non-verbal cues and group dynamics.

### Data analysis

Audio recordings were transcribed verbatim and translated into English for analysis. Thematic analysis was performed following Braun and Clarke’s (2006) six-phase framework.^9^ This involved an initial phase of data familiarisation, followed by manual coding of transcripts to identify descriptive and interpretative elements. Codes were then organised into broader themes, informed by both a priori categories and inductively derived insights. Themes were reviewed for internal coherence and distinctiveness, and subthemes were defined using consistent operational criteria. Representative participant quotations were selected to illustrate each theme, ensuring contextual clarity and authenticity.

Two independent researchers performed parallel coding to enhance the reliability and validity of theme development. Discrepancies in thematic assignment were discussed and reconciled collaboratively, enhancing intercoder reliability and analytical validity. Thematic saturation was monitored concurrently with data collection and was defined as the point at which no new themes or subthemes emerged in subsequent FGDs. After eight FGDs, data began to demonstrate significant redundancy, with recurring narratives and concerns being consistently reported across different groups. The final two FGDs were used to validate and confirm the thematic stability, thereby affirming saturation.

To enhance trustworthiness and respondent validation, preliminary thematic summaries were shared with a purposively selected subgroup of participants (n=5) representing different geographic subareas within the two districts. Feedback was sought regarding the resonance and accuracy of interpretations. Respondents endorsed the validity of the themes and offered minor clarifications, which were incorporated into the final analysis. Due to logistical limitations, full transcript returns were not performed; however, this targeted member checking process provided adequate triangulation to bolster analytic credibility.

### Ethical considerations

Ethical clearance was granted by the Ethics Review Committee of the Postgraduate Institute of Medicine, University of Colombo (Reference: ERC/PGIM/2024/080). All participants were provided with detailed study information in their native language and gave written informed consent prior to participation. Confidentiality was safeguarded through the anonymisation of transcripts and secure data storage accessible only to the research team. No financial incentives were offered; however, light refreshments were provided as a gesture of appreciation.

### Patient and public involvement

Patients and/or the public were not involved in the design, or conduct, or reporting or dissemination plans of this research.

## Results

### Participant characteristics

A total of 70 parents participated in focused group discussions (table 1).

**Table 1:** Personal characteristics of the study population

Characteristic	Number (N=70)	Per cent
Sex of the participants		
Male	25	48.8
Female	45	51.2
Age		
20–24	17	34.3
25–29	28	40.0
30–34	16	22.8
35–39	4	5.7
40–44	3	4.3
44–49	2	2.9
Highest education level in the parent		
Did not pass GCE ordinary level (O/L)	9	12.9%
Passed GCE ordinary level (O/L)	35	50.0%
Passed GCE advanced level (A/L)	19	27.1%
Degree or higher	5	7.1%
Monthly household income (LKR)		
Less than 10 000	2	2.8%
10 000–24 999	10	14.3%
25 000–49 999	10	14.3%
50 000–99 999	44	62.9%
More than 100 000	4	5.7%
District		
Polonnaruwa	34	48.6
Ampara	36	51.4

A/L, Advanced level; GCE, General Certificate in Education; O/L, Ordinary Level.

Thematic analysis of FGDs with 70 parents living in rural Sri Lanka revealed a complex interplay of fear, fragmented knowledge, environmental exposure, traditional beliefs and aspirations for education in the context of paediatric snakebites. Participants—predominantly mothers—shared lived experiences that illuminated not only the physical risks associated with snakebite but also the psychosocial and systemic dimensions of prevention and response. Five core themes emerged from the discussions: (1) constant fear and psychological burden, (2) limited and inconsistent knowledge of snakebite symptoms and first-aid, (3) environmental and structural vulnerabilities, (4) traditional beliefs and treatment practices and (5) a strong desire for education and systemic support.

A persistent and deeply rooted fear of snakebite dominated parental narratives, particularly with regard to their young children. Most parents viewed snakebite as a sudden, unpredictable and life-threatening event, with potentially fatal consequences. The emotional toll of this fear was significant. Some parents described chronic anxiety, particularly during the rainy season or at night, when snakes were thought to be more active. *Even if we clean the garden, snakes still come*, said one mother (parent 24). *We are scared to let the children go out in the evening* (parent 33). Others echoed similar fears, stating, *My child is only two years old. He plays in the garden, and we’ve seen cobras there. I am always watching him*. (parent 54). The psychological burden was compounded by the recognition that children may not always be able to identify or report snakebites. One parent recounted, *My child was very small so she couldn’t even tell us what happened. We feared and rushed to the hospital*. In many households, even after a bite incident, lingering trauma was reported, with children avoiding the site of the bite or expressing persistent pain and fear long after recovery.

Although parents had a general understanding that snakebites could be dangerous, their knowledge of identifying venomous snakes and recognising symptoms of envenomation was variable and often incomplete. Some participants described observing bite marks, swelling, bleeding or unconsciousness. *The bite site becomes painful and swollen*, one mother noted (parent 08). *The child may vomit or even lose consciousness* (parent 37). However, several others admitted uncertainty. *I have no idea about identifying poisonous snakes*, said one participant (parent 64). *I just know if the child vomits or faints, it’s serious* (parent 35). Descriptions of specific symptoms included numbness, blurred vision, lethargy, difficulty breathing and blackish discolouration at the bite site. A few participants attempted to distinguish between venomous species based on appearance—*Krait has shiny black skin with white bands* (parent 33)—but others emphasised their inability to correctly identify snakes, especially when bites occurred at night. Notably, several parents reported that their children had been bitten while playing in grassy or bushy areas, while walking to outdoor toilets, or when handling firewood or garbage. One child was bitten while trying to retrieve a ball, another while bathing in the river and another while playing near a pile of bricks.

First-aid practices were highly inconsistent and often included both helpful and potentially harmful methods. The most common initial responses included washing the bite site, applying soap or lemon, tying a cloth tightly above the wound and rushing to the nearest hospital. *We washed the area and tied a piece of cloth*, one parent shared (parent 27). *Then we brought him on a bike to the hospital* (parent 27). Others emphasised the need to keep the victim calm and immobilised. However, incorrect practices were also frequently reported. Tourniquets, tight bindings and herbal applications were described as inherited methods passed down through generations. *We tied the leg tightly with a woman’s hair,* said one mother (parent 42). *It stops the poison from moving up* (parent 42). In many cases, these actions were performed before any consultation with medical professionals. A small number of participants, particularly those who worked in healthcare, demonstrated better knowledge of evidence-based first-aid protocols, but such examples were rare. Several participants also revealed that first-aid knowledge was acquired from family elders or learnt informally through social networks, YouTube videos, or past experience. Formal first-aid training was almost universally lacking.

Several participants recounted strong cultural beliefs related to snakes and their divine associations. A father explained, *The cobra is a sacred animal and is connected to God Shiva. It also lives around Bodhi trees. Killing a cobra is not just a bad omen—it brings consequences* (parent 33). Others linked snake encounters with spiritual or moral consequences. A participant recalled a family incident: *My father tried to kill a cobra and was bitten. He had paralysis and couldn’t see properly for three months. He recovered, but we believe the attack was a punishment. That snake was eventually killed* (parent 47). One parent shared a personal experience that emphasised the need for caution: *My husband saw a snake near the shed and thought it might be dangerous to our children, so he took an axe to kill it. But when he struck, the axe bounced and hit his arm. He had to get stitches. Later, they said the snake wasn’t even venomous* (parent 30). This highlights the importance of snake identification and safe approaches in responding to snake encounters. Additionally, some believed that a snake becomes weak or loses its strength after biting a human: *Snakes lose their power after biting, that’s why they go away or die afterward* (parent 21). These perspectives influenced both preventive behaviours and first-aid responses, often guiding decisions on whether to seek biomedical care or rely on rituals and traditional practices.

The physical environment in which these families lived significantly contributed to the risk of snakebite. Most participants reported frequent sightings of snakes around their homes, particularly in firewood piles, coconut husks, dry leaf collections and abandoned lands. Termite mounds, outdoor toilets, paddy fields and irrigation canals were also identified as high-risk areas. One participant noted, *Snakes hide in the coconut shells and husks near the firewood pile. That’s where the bite happened* (parent 22). Another added, *They come from the jungle and cross the road near our house. My daughter was bitten when stepping outside to pick flowers* (parent 32). During the rainy season, snake sightings and bites reportedly increased. In several households, snakes had entered living spaces, with one child reportedly bitten in bed and another in the kitchen. Despite attempts to mitigate risk by cleaning gardens and burning leaf litter, structural vulnerabilities persisted. Families lacked protective infrastructure such as indoor toilets, sealed housing or raised beds. Several described pouring kerosene oil around the house or burning chilli to deter snakes. Some had built small fences or walls, but many noted that neighbouring lands remained overgrown and unsafe, limiting the effectiveness of individual efforts.

Traditional beliefs and practices played a significant role in shaping how parents interpreted and responded to snakebites. Cultural narratives surrounding cobras and kraits were particularly prevalent. Many participants believed that killing a cobra without proper rituals could bring misfortune. *If you kill a cobra and don’t burn it, seven more will come for revenge*, said one father (parent 25). Others described religious offerings made after sightings or bites, including boiling coconut milk, tying vow knots and performing temple rituals. A belief that speaking the name of the snake after a bite worsened the poisoning was shared in multiple groups. Local healing practices included the use of crushed herbs, oil-based treatments, chanting and the application of *visha gala* snake stones believed to extract venom. *They placed a special stone on the bite. If it sticks, it means the poison is being pulled out,* one participant explained (parent 26). While some parents expressed scepticism, others emphasised that such practices were used in desperation, especially when transport to hospitals was delayed. One participant reflected, *I don’t believe in all this, but when the hospital is far, you try everything* (parent 24)

Despite significant challenges, participants consistently expressed a strong desire for accurate information, skill-based training and preventive education. Many parents emphasised the need for community-wide programmes tailored to local contexts. *We need someone to come and teach us again and again*, one mother said (parent 42). *Just one session is not enough*. Participants preferred education through schools, village meetings and religious gatherings, with practical demonstrations and pictorial materials. Parents specifically requested training on how to identify dangerous snakes, administer proper first aid and prevent bites. *Teach us how to check firewood, how to tie a band, and when to go to the hospital*, said one father (parent 44). Some participants proposed distributing torches, gumboots and safety tools, while others called for government assistance in improving household infrastructure. Health system barriers—such as transport challenges, long distances to hospital, lack of antivenom and poor staffing—were repeatedly cited as impediments to timely care. *We live 25 kilometres from the hospital*, said one parent. *At night, there are elephants on the road. What do we do if a child is bitten then?* (parent 55)

### Second-order analysis

Beyond the descriptive patterns identified, the findings reflect a deeper sociocultural tension between traditional ecological knowledge and biomedical frameworks of snakebite prevention and treatment. Parents’ reliance on inherited practices—such as the application of snake stones, tight tourniquets and ritualistic offerings—signifies not merely a lack of access to formal health education, but a form of situated expertise rooted in intergenerational experience and collective memory. While many of these practices may be ineffective or harmful from a medical standpoint, they serve an important symbolic function: reducing uncertainty, asserting control in emergencies and preserving cultural continuity in the face of perceived threats. This suggests that educational interventions should not aim to displace these practices through top-down correction but instead engage respectfully with local belief systems, coproducing knowledge in ways that foster trust and sustainable behaviour change.

Additionally, the parents’ narratives reveal how structural violence—manifested in poverty, poor infrastructure and healthcare inaccessibility—shapes the context in which snakebites occur and are managed. Their ‘choices’ in first-aid and transport are not entirely voluntary but constrained by spatial isolation, economic hardship and ecological exposure. The fear expressed by parents is not only of the snake itself but also of the systemic failure to protect their children in time. In this light, the community’s perceived vulnerability can be understood not merely as a deficit of knowledge, but as a rational response to an under-resourced environment. The findings further reveal the importance of multisectoral interventions that address not only knowledge and attitudes but also the broader social determinants that perpetuate paediatric snakebite risk in rural Sri Lanka. Parents often show a strong willingness to learn, engage and improve their response if supported by culturally appropriate, accessible and sustained interventions. In this regard, the lived experiences and insights of parents are crucial for informing community-based strategies to reduce the burden of paediatric snakebites in Sri Lanka.

## Discussion

This study illuminates the complex interplay of fear, cultural beliefs, environmental vulnerabilities and systemic healthcare limitations that shape parental responses to paediatric snakebites in rural Sri Lanka. The findings underscore the necessity for culturally sensitive, community-based interventions to address both the immediate and underlying factors contributing to snakebite morbidity and mortality among children. A pervasive theme among participants was the chronic anxiety surrounding snakebite incidents, particularly concerning their children’s safety. This heightened vigilance often led to behavioural modifications, such as restricting children’s outdoor activities. Such psychological burdens are consistent with findings from other high-incidence regions, where the fear of snakebites significantly impacts daily life and mental health.^10^ Despite recognising snakebites as a serious health threat, many parents exhibited inconsistent knowledge regarding venomous species, symptom identification and appropriate first-aid measures. Traditional practices, such as applying tourniquets or using herbal remedies, remain prevalent, reflecting a knowledge-practice mismatch previously documented among Sri Lankan farmers.^10^ This discrepancy highlights the need for targeted educational programmes that bridge the gap between awareness and practice.^11^

Cultural and religious beliefs significantly influence how snakebites are interpreted and managed.^12^ For instance, cobras are often regarded as sacred beings associated with divine retribution, leading to rituals like burning the snake’s body to prevent further danger. These beliefs can delay or deter seeking biomedical care, as traditional interpretations may prioritise spiritual interventions over medical treatment. Engaging with these cultural frameworks is crucial for public health initiatives to be effective and respectful of local traditions.^13^

Environmental and structural vulnerabilities exacerbate the risk of paediatric snakebites. Unsafe housing conditions, inadequate waste disposal and proximity to snake habitats increase exposure, particularly during seasonal peaks in snake activity.^14^ Implementing structural improvements, such as safe toilets and sealed flooring, alongside behavioural education, can mitigate these risks.

Participants expressed a strong desire for education, practical training and government support. Their openness to community programmes suggests that behaviour change is attainable when interventions are collaboratively designed, culturally appropriate and address material realities. Preferred delivery channels included schools, religious gatherings and mobile health units, indicating the potential for multisectoral collaboration. Systemic healthcare gaps, including delays in treatment due to transport challenges and limited availability of antivenom, were frequently cited. These delays are not solely due to cultural preferences but are also a consequence of material deprivation and infrastructural inadequacies.^15^ Addressing these systemic issues is essential for improving outcomes in paediatric snakebite cases.

This study has several limitations. First, it was geographically limited to two high-incidence districts in Sri Lanka, which may restrict generalisability to other regions with different cultural or ecological profiles. Second, the study did not include direct observation of household environments or follow-up interviews to triangulate findings, which could have enriched contextual understanding. Finally, fathers were less represented than mothers, potentially skewing perspectives on family decision-making and caregiving roles. Despite these limitations, the study provides valuable qualitative insights into the lived experiences of caregivers managing paediatric snakebite risk.

## Conclusion

Paediatric snakebites in rural Sri Lanka are not merely a medical emergency but a deeply embedded sociocultural and structural issue. This study underscores the complex interplay of fear, cultural beliefs, environmental exposure and health system limitations that shape parental responses to snakebite incidents. While many practices are influenced by tradition, there is a strong willingness among parents to adopt evidence-based prevention and first-aid strategies when supported through culturally appropriate and accessible education. Addressing paediatric snakebite requires not only knowledge dissemination but also investments in healthcare infrastructure, transport systems and household safety. Collaborative, community-led interventions that honour local belief systems while promoting safe practices offer the most sustainable path towards reducing the burden of paediatric snakebite in Sri Lanka.

## Data Availability

Data are available upon reasonable request.
