# Supplementary material for: Caught between fear and tradition: parental knowledge, beliefs and emergency responses to paediatric snakebites in rural Sri Lanka
Source: BMJ Paediatr Open. 2025 Jun 13;9(1):e003658. doi: 10.1136/bmjpo-2025-003658 (PMC12164603; doi:10.1136/bmjpo-2025-003658)
Supplement: Supplementary file 1 [file bmjpo-2025-003658.reviewer_comments.pdf]

PEER REVIEW HISTORY

BMJ Paediatrics Open publishes all reviews undertaken for accepted manuscripts. Reviewers are asked to complete a checklist review form and are provided with free text boxes to elaborate on their assessment. These free text comments are reproduced below.

ARTICLE DETAILS

**TITLE (PROVISIONAL)**	Caught between fear and tradition: parental knowledge, beliefs, and emergency responses to paediatric snakebites in rural Sri Lanka
**AUTHORS**	Dayasiri, Kavinda; Perera, Tharuka; Gawarammana, Indika; Jayamanne, Shaluka

VERSION 1 - REVIEW

**REVIEWER NAME**	***Deb Pandey***
**REVIEWER AFFILIATION**	Agriculture and Forestry University, Department of Veterinary Microbiology and Parasitology
**REVIEWER CONFLICT OF INTEREST**	N/A
**AI DISCLOSURE**	No
**DATE REVIEW RETURNED**	22-May-2025

**GENERAL COMMENTS**	bmjpo-2025-003658_Proof_hi<br>General<br>Authors have nicely presented the interplay of fear, cultural beliefs, environmental exposure, and health system limitations regarding the paediatric snakebites in rural Sri Lanka where snakebite is sociocultural and structural issues. Despite limitations (which they clearly illustrated), this study has significant implication in prevention and prehospital care of snakebite in Sri Lanka. I would suggest accepting this article with a couple of nominal improvements suggested below.<br>Specific<br>Please, replace "Ampara and Polonnaruwa districts" with Ampara and Polonnaruwa Districts. It is because common names are capitalized.<br>Please, be consistent in the use of first aid or first-aid.

**REVIEWER NAME**	***Geetha Anand***
**REVIEWER AFFILIATION**	Oxford University Hospitals NHS Foundation Trust
**REVIEWER CONFLICT OF INTEREST**	I know one of the authors
**AI DISCLOSURE**	No
**DATE REVIEW RETURNED**	27-May-2025

**GENERAL COMMENTS**	This is a well written paper on a topic that is most relevant to the setting.<br>Direct quotes from parents make it more readable and real.<br>The focus on paediatric snakebite in rural Sri Lanka fills a notable gap in global health literature, particularly on how sociocultural dynamics influence emergency responses.

VERSION 1 – AUTHOR RESPONSE

Reviewer: 1

Dr. Deb Pandey, Agriculture and Forestry University

General

Authors have nicely presented the interplay of fear, cultural beliefs, environmental exposure, and health system limitations regarding the paediatric snakebites in rural Sri Lanka where snakebite is sociocultural and structural issues. Despite limitations (which they clearly illustrated), this study has significant implication in prevention and prehospital care of snakebite in Sri Lanka. I would suggest accepting this article with a couple of nominal improvements suggested below.

Specific

Please, replace "Ampara and Polonnaruwa districts" with Ampara and Polonnaruwa Districts. It is because common names are capitalized.

Thank you very much!! We have revised as advised in both the abstract and the body of the manuscript.

Please, be consistent in the use of first aid or first-aid.

Thank you very much!! We have revised as advised. We have now consistently mentioned this as “first-aid” in both the abstract and the body of the manuscript.

Reviewer 2

Geetha Anand, Oxford University Hospitals NHS Foundation Trust

Comments to the Author

This is a well written paper on a topic that is most relevant to the setting. Direct quotes from parents make it more readable and real. The focus on paediatric snakebite in rural Sri Lanka fills a notable gap in global health literature, particularly on how sociocultural dynamics influence emergency responses.

Thank you very much for the comment!!
